# Inter‐ and intraspecific variation of spider mite susceptibility to fungal infections: Implications for the long‐term success of biological control

**DOI:** 10.1002/ece3.5958

**Published:** 2020-03-06

**Authors:** Flore Zélé, Mustafa Altıntaş, Inês Santos, Ibrahim Cakmak, Sara Magalhães

**Affiliations:** ^1^ Centre for Ecology, Evolution and Environmental Changes (cE3c) Faculdade de Ciências Universidade de Lisboa Lisboa Portugal; ^2^ Department of Plant Protection Faculty of Agriculture Adnan Menderes University Aydin Turkey

**Keywords:** entomopathogenic fungi, host evolution, parasite‐induced mortality, resistance, Tetranychidae, tolerance

## Abstract

Spider mites are severe pests of several annual and perennial crops worldwide, often causing important economic damages. As rapid evolution of pesticide resistance in this group hampers the efficiency of chemical control, alternative control strategies, such as the use of entomopathogenic fungi, are being developed. However, while several studies have focused on the evaluation of the control potential of different fungal species and/or isolates as well as their compatibility with other control methods (e.g., predators or chemical pesticides), knowledge on the extent of inter‐ and intraspecific variation in spider mite susceptibility to fungal infection is as yet incipient. Here, we measured the mortality induced by two generalist fungi, *Beauveria bassiana* and *Metarhizium brunneum*, in 12 spider mite populations belonging to different *Tetranychus* species: *T. evansi*, *T. ludeni*, and *T. urticae* (green and red form), within a full factorial experiment. We found that spider mite species differed in their susceptibility to infection by both fungal species. Moreover, we also found important intraspecific variation for this trait. These results draw caution on the development of single strains as biocontrol agents. Indeed, the high level of intraspecific variation suggests that (a) the one‐size‐fits‐all strategy may fail to control spider mite populations and (b) hosts resistance to infection may evolve at a rapid pace. Finally, we propose future directions to better understand this system and improve the long‐term success of spider mite control strategies based on entomopathogenic fungi.

## Graphical abstract

This study shows that spider mite species, and populations within the same species, differed in their susceptibility to infection by entomopathogenic fungi. Because variation in host susceptibility to infection constitutes potential for evolution of resistance, these results draw caution on the development of single strains as biocontrol agents and highlight the importance of evaluating the susceptibility of multiple host populations/genomes when assessing the efficiency of biocontrol agents.

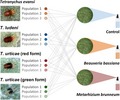

## 1 INTRODUCTION

Pesticides are still the main weapon used to control crop pests and disease vectors, despite the major threats they represent for food safety and for the environment (Bourguet & Guillemaud, 2016). Moreover, the pervasive evolution and rapid spread of resistance to pesticides severely affect their efficiency in many taxa (Casida & Quistad, 1998). Therefore, alternative control strategies are being sought to control disease epidemics and outbreaks of agricultural crop pests (Hajek, McManus, & Delalibera, 2007; Lacey, Frutos, Kaya, & Vail, 2001; Parolin et al., 2012; Zindel, Gottlieb, & Aebi, 2011), including spider mites (Attia et al., 2013).

Spider mite of the genus *Tetranychus* (Acari: Tetranychidae) are ubiquitous major crop pests of c.a. 1,100 plant species belonging to more than 140 different plant families (Migeon & Dorkeld, 2006–2017–2017), destroying annual and perennial crops. A few studies have evaluated the economic costs of spider mites, which vary among crops, seasons, and plant age (Alatawi, Margolies, & Nechols, 2007; Flamini, 2006; Opit, Chen, Williams, Nechols, & Margolies, 2005; Park & Lee, 2002, 2005, 2007; Weihrauch, 2005), and theoretical studies suggest that detrimental effects of spider mites in agriculture will dramatically increase with increased global warming (Migeon et al., 2009). Moreover, due to their short generation time and high fecundity, spider mites rapidly develop resistance to pesticides (Van Leeuwen, Vontas, Tsagkarakou, Dermauw, & Tirry, 2010). Important efforts are thus being developed to evaluate the efficiency of different biological control methods, such as the use of essential oils or natural enemies (e.g., predators, entomopathogenic bacteria, and fungi; Attia et al., 2013). In particular, a plethora of studies have evaluated the virulence of many fungal species (e.g., *Neozygites* spp., *Metarhizium* spp., *Beauveria bassiana*, and *Lecanicillium lecanii*) and/or strains to identify the best candidates for efficient spider mite control (e.g., Bugeme, Maniania, Knapp, & Boga, 2008; Chandler, Davidson, & Jacobson, 2005; Maniania, Bugeme, Wekesa, Delalibera, & Knapp, 2008; Shi, Zhang, & Feng, 2008; Shin, Bae, Kim, Yun, & Woo, 2017), as well as their compatibility with other control methods, such as predatory mites (e.g., Dogan, Hazir, Yildiz, Butt, & Cakmak, 2017; Seiedy, 2014; Seiedy, Saboori, & Allahyari, 2012; Seiedy, Saboori, & Zahedi‐Golpayegani, 2013; Ullah & Lim, 2017; Vergel, Bustos, Rodriguez, & Cantor, 2011; Wu, Xie, Li, Xu, & Lei, 2016) or pesticides (e.g., Irigaray, Marco‐Mancebon, & Perez‐Moreno, 2003; Klingen & Westrum, 2007; Shi, Jiang, & Feng, 2005). However, these studies were conducted using a single host population and potential intraspecific variations in spider mites susceptibility have, to our knowledge, never been investigated within a single experiment (but see, for instance: Afifi, Mabrouk, & Asran, 2010, Fiedler & Sosnowska, 2007, Ribeiro, Gondim, Calderan, & Delalibera, 2009 for a comparison among spider mites and/or among other arthropod species; or Milner, 1982, 1985, Perinotto et al., 2012, Uma Devi, Padmavathi, Uma Maheswara Rao, Khan, & Mohan, 2008 for intraspecific variation within other arthropod species).

Both intra‐ and interspecific variability in host susceptibility to infection may modify epidemiological patterns of parasite in natural host populations (Dwyer, Elkinton, & Buonaccorsi, 1997; Hawley & Altizer, 2011; Read, 1995), thereby altering the efficiency and environmental persistence of biocontrol agents. Moreover, the use of such agents generates a strong selection pressure on the target pests (e.g., Fenner & Fantini, 1999, see also Tabashnik, 1994, Moscardi, 1999) and, in general, variability in host susceptibility to infection may have important consequences for the evolution of host resistance as well as parasite virulence and transmission (Elena, 2017; Sorci, Moller, & Boulinier, 1997; Stevens & Rizzo, 2008). Hence, assessing both intra‐ and interspecific variability in spider mite susceptibility to infection by different potential biocontrol agents is a prerequisite for the development of efficient and long‐lasting control strategies.

To this aim, we assessed the susceptibility to fungi infection of 12 different spider mite populations belonging to different species that are ubiquitous in Europe and often co‐occur in the field (Migeon & Dorkeld, 2006–2017–2017, Zélé, Santos, Godinho, & Magalhães, 2018b): three populations of the green form of *T. urticae*, three populations of the red form of *T. urticae* (also referred to as *T. cinnabarinus* by some authors; e.g., Shi & Feng, 2004, Shi et al., 2005, Li, Chen, & Hong, 2009), three populations of *T. ludeni*, and three populations of *T. evansi*. We used two generalist entomopathogenic fungi species, *B. bassiana *and *Metarhizium brunneum*, as *Beauveria* and *Metarhizium* spp. are among the most used fungi in commercial production (Vega et al., 2009), and have wide geographical and host ranges (Greif & Currah, 2007; Gurlek, Sevim, Sezgin, & Sevim, 2018; Meyling & Eilenberg, 2007; Rehner, 2005; Roberts & Leger, 2004). We then discuss the possible ecological and evolutionary causes and underlying mechanisms leading to the observed results, as well as their potential consequences for the evolution of both hosts susceptibility to infection and fungi virulence. Finally, we propose future directions to improve long‐term success of spider mite control strategies using entomopathogenic fungi.

## 2 MATERIALS AND METHODS

### 2.1 Spider mite populations and rearing

Twelve populations of Tetranychid mites were used in this study: three of *T. evansi* (called BR, GH, and QL), three of *T. ludeni* (called OBI, Alval, and Assaf), three of the red form of *T. urticae* (called AlRo, AMP.tet, and FR.tet), and three of the green form of *T. urticae* (called TOM.rif, LS.tet, and B6JS). Most of these populations were collected in Portugal from 2013 to 2016; FR.tet was collected in France and AlRo in Spain in 2013. The population BR of *T. evansi* was collected in a greenhouse in Brazil in 2002 (Godinho, Janssen, Dias, Cruz, & Magalhães, 2016; Sarmento et al., 2011), and the population LS.tet of the green form of *T. urticae* derived from the London strain, which was used to sequence the species genome (Grbic et al., 2011). These populations originated from various plant species in the field, and none of them carried bacterial endosymbionts (i.e., *Wolbachia*, *Cardinium*, *Rickettsia*, *Arsenophonus*, *Spiroplasma*), either because they were initially uninfected when collected in the field (Zélé, Santos, Olivieri, et al., 2018a), or following antibiotic treatment (three generations with tetracycline hydrochloride, or one generation with rifampicin; all populations with “.tet” or “.rif” suffix, respectively; Breeuwer, 1997; Gotoh et al., 2005; Li, Floate, Fields, & Pang, 2014). All the information concerning these populations is summarized in (Table 1). They were subsequently reared in the laboratory under standard conditions (25 ± 2°C, 60% RH, 16/8‐hr L/D) at high numbers (c.a. 500–1,000 females per cage) in insect‐proof cages containing either bean cv. Contender seedlings (obtained from Germisem, Oliveira do Hospital, Portugal) for *T. urticae* and *T. ludeni*, or tomato cv. Money Maker seedlings (obtained from Mr. Fothergill's Seeds, Kentford, UK) for *T. evansi*.

**Table 1:** Populations of spider mites used in the experiment

Species	Name	Date	Host plant	Location	Coordinates	Antibiotics	Reference
*Tetranychus evansi*	BR	‐‐/‐‐/2002	*Solanum lycopersicum*	Unknown (B)	Unknown	–	Sarmento et al. (2011)
	GH	03/10/2013	*Solanum lycopersicum*	University of Lisbon (P)	38.757852, −9.158221	–	Zélé, Santos, Olivieri, et al. (2018a)
	QL	26/11/2013	*Datura stramonium*	Quinta das Lameiras (P)	39.085837, −8.991478	–	Zélé, Santos, Olivieri, et al. (2018a)
*Tetranychus ludeni*	OBI	09/10/2016	*Ipomoea purpurea*	Óbidos, Gracieira (P)	39.334049,−9.121178	–	–
	Alval	05/11/2013	*Ipomoea purpurea*	Alvalade, Lisbon (P)	38.75515, −9.14685	–	Zélé, Santos, Olivieri, et al. (2018a)
	Assaf	20/09/2013	*Datura stramonium*	Assafora (P)	38.904743, −9.408592	–	Zélé, Santos, Olivieri, et al. (2018a)
*Tetranychus urticae* red form	AlRo	09/11/2013	*Rosa spp.*	Almería (S)	36.855725, −2.320374	–	Zélé, Santos, Olivieri, et al. (2018a)
	AMP.tet	18/11/2013	*Datura stramonium*	Aldeia da Mata Pequena (P)	38.534363, −9.191163	13/01/2013^a^	Zélé, Santos, Olivieri, et al. (2018a)
	FR.tet	11/10/2013	*Solanum lycopersicum*	Montpellier (F)	43.614951, 3.859846	26/12/2013^a^	Zélé, Santos, Olivieri, et al. (2018a)
*Tetranychus urticae* green form	TOM.rif	‐‐/05/2010	*Solanum lycopersicum*	Carregado (P)	39.078962,−8.993656	15/09/2016^b^	Clemente, Rodrigues, Ponce, Varela, and Magalhães (2016)
	LS.tet	Unknown	*Phaseolus vulgaris*	Vineland, Ontario (C)	Unknown	13/01/2013^a^	Grbic et al. (2011)
	B6JS^c^	10/06/2015	*Phaseolus vulgaris*	Correias (P)	39.342914, −8.797936	–	Zélé, Santos, Godinho, et al. (2018b)

Note

Mites were collected in Canada (C), Brazil (B), Portugal (P), France (F), and Spain (S). Populations harboring bacterial endosymbionts (e.g., *Wolbachia*, *Cardinium*, *Rickettsia*) were cured from infection with antibiotics.

a Tetracycline hydrochloride (Breeuwer, 1997)

b Rifampicin (Gotoh et al., 2005)

c Population called B6 in the supplementary materials of Zélé, Santos, Godinho, et al. (2018b)

### 2.2 Entomopathogenic fungi strains and preparation of inoculum

We used the strains V275 (=Met52, F52, BIPESCO 5) of *M. brunneum* and UPH‐1103 of *B. bassiana *, obtained from Swansea University (UK) and from Siedlce University (Poland), respectively, as they were previously shown to have the potential to suppress *T. urticae* populations (Dogan et al., 2017). The procedures used for fungal growth, inoculum preparation, and spider mite infection are similar to that described in Dogan et al. (2017). Briefly, the two fungi were grown on Sabouraud Dextrose Agar (SDA) medium at 25°C for 2 weeks. Conidia were harvested from sporulating cultures with the aid of a spatula, washed with sterile distilled water, and filtered through four layers of gauze to remove any hyphae.

### 2.3 Spider mite infection and survival

The experiment was conducted in a growth chamber under standard conditions (25 ± 2°C, 80% RH, 16/8‐hr L/D). Roughly 2 weeks prior to the experiment, age cohorts were created for each spider mite population by collecting ca. 100 females from each mass culture, allowing them to lay eggs during 4 days on detached bean leaves placed on water‐soaked cotton. The offspring from these cohorts was used in the experiment.

One day prior to the onset of this experiment, 20 adult mated females with similar age were randomly collected from each cohort and placed on a 9‐cm^2^ bean leaf disk on top of wet cotton (to ensure the leaf remained hydrated) with the abaxial (underside) surface facing upwards. On the first day of the experiment, the surface of the leaf disks was sprayed using a hand sprayer with 2.5 ml of a spore suspension of *M. brunneum* or *B. bassiana *in 0.03% (v/v) aqueous Tween‐20 at 1 × 10^7^ conidia/ml, or, as control, with 0.03% aqueous Tween‐20 only. Subsequently, female survival was monitored every 24 hr during 10 days by counting both dead and alive individuals. A total of twelve replicates per infection treatment (sprayed with *B. bassiana *, with *M. brunneum*, or with Tween‐20 only) per population of each species were performed within seven temporal blocks (roughly three replicates of each treatment per block).

### 2.4 Statistical analysis

The analyses were carried out using the R statistical package (version 3.5.3). Survival data were analyzed using Cox proportional hazards mixed‐effect models (coxme, kinship package). Spider mite species, or populations within each species, and infection treatment (sprayed with *B. bassiana *, with *M. brunneum*, or with Tween‐20 only as control) were fit in as fixed explanatory variables, whereas disks nested within population, population (in the case of interspecific variation only), and block were fit as random explanatory variables. Hazard ratios (HR) were obtained from these models as an estimate of the difference between the rates of dying (i.e., the instantaneous rate of change in the log number of survivors per unit of time; Crawley, 2007) between the controls of each species/population (by changing the intercept of the model) and the *B. bassiana* or *M. brunneum* treatments.

Maximal models, including all higher‐order interactions, were simplified to establish a minimal model by sequentially eliminating nonsignificant terms and interactions (Crawley, 2007). The significance of the explanatory variables was established using chi‐squared tests (Bolker, 2008). The significant chi‐squared values given in the text are for the minimal model, whereas nonsignificant values correspond to those obtained before deletion of the variable from the model.

To further explore significant interactions between species/population and treatment effects on female survival, the two factors were concatenated to fit a single fixed factor containing all species/population by treatments levels in the models (i.e., 12 levels for species by treatment effects, or 9 levels for population by treatment effects within each species). Multiple comparisons between levels were then performed from these models using general linear hypotheses (glht, package multicomp) with Holm corrections, which uses classical chi‐square (Wald test) for testing the global hypothesis *H*_0_.

## 3 RESULTS

### 3.1 Interspecific variation of spider mite susceptibility to infection by *Beauveria bassiana* and *Metarhizium brunneum*

**Figure 1:**
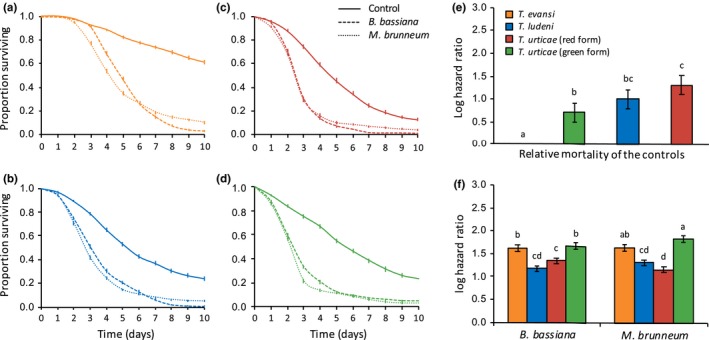
Survival curves of spider mite females (proportion surviving ± *SE*) from (a) *Tetranychus evansi*, (b) *Tetranychus ludeni*, (c) red form of *Tetranychus urticae*, and (d) green form of *T. urticae*, sprayed with *Beauveria bassiana* (dashed lines), *Metarhizium brunneum* (dotted lines), or with Tween‐20 only (control; solid lines). (e) Estimated mortality of the controls (sprayed with Tween‐20 only) of each spider mite species relative to the control of *T. evansi* (log hazard ratio ± *SE*). (f) Estimated mortality of each spider mite species upon infection by each fungus relative to their respective control (log hazard ratio ± *SE*); identical letter superscripts indicate nonsignificant differences between treatments at the 5% level (multiple comparisons with Holm correction)

The statistical analyses revealed a significant interaction between treatments (females sprayed with either Tween‐20 only as control, *B. bassiana *, or *M. brunneum*) and species (*T. evansi*, *T. ludeni*, red and green form of *T. urticae*) on the survival of spider mites (*X* ^2^_6_ = 80.61, *p* < .001; Figure 1). Indeed, multiple comparisons of hazard ratios (HRs) revealed that all spider mite species were not equally affected by infection (Figure 1e; Table 2 for the statistical results of all multiple comparisons): Both fungi induced a stronger mortality in *T. evansi* (HR = 5.04 for *B. bassiana *, and HR = 5.15 for *M. brunneum*) and in the green form of *T. urticae* (HR = 5.30 for *B. bassiana *, and HR = 6.27 for *M. brunneum*), than in *T. ludeni* (HR = 3.25 for *B. bassiana *, and HR = 3.73 for *M. brunneum*) and in the red form of *T. urticae* (HR = 3.84 for *B. bassiana *, and HR = 3.21 for *M. brunneum*). Moreover, while the two fungi induced similar mortality in *T. evansi* and in *T. ludeni*, infection with *B. bassiana* led to higher mortality than *M. brunneum* in the red form of *T. urticae*, while the reverse was found in the green form of *T. urticae*. Note, however, that survival in the *T. evansi* control was higher than in that of the three other species (Figure 1d and Table 2).

**Table 2:** Results of multiple comparisons (with Holm correction) between hazard ratios obtained for different spider mite species (Te: *Tetranychus evansi*; Tl: *Tetranychus ludeni*; TuR: red form of *Tetranychus urticae*; TuG: green form of *Tetranychus urticae*) among the different treatments (BB: *Beauveria bassiana*; MB: *Metarhizium brunneum*; C: Control)

Type of comparison	Species_treatments compared	Estimate	*SE*	*z* value	*p*‐value
Between controls	Te_C versus TuG_C	−0.710	0.204	−3.472	.008**
	TuG_C versus Tl_C	−0.288	0.202	−1.427	.768
	Tl_C versus TuR_C	−0.303	0.196	−1.541	.740
	TuG_C versus TuR_C	−0.590	0.201	−2.934	.040*
Between species sprayed with *B. bassiana *	TuG_BB versus Te_BB	0.051	0.097	0.523	1.000
	TuR_BB versus Te_BB	−2.272	0.095	−2.866	.046*
	TuR_BB versus Tl_BB	0.166	0.086	1.927	.432
Between species sprayed with *M. brunneum*	TuG_MB versus Te_MB	0.196	0.097	2.011	.399
	Tl_MB versus Te_MB	−0.323	0.097	−3.337	.013*
	TuR_MB versus Tl_MB	−0.149	0.088	−1.695	.630
Between fungi within or among species	Te_BB versus Te_MB	−0.022	0.056	−0.391	1.000
	Tl_BB versus Tl_MB	−0.137	0.055	−2.481	.131
	TuR_BB versus TuR_MB	0.179	0.057	3.128	.025*
	TuG_BB versus TuG_MB	−0.167	0.056	−2.961	.040*
	TuG_BB versus Te_MB	0.029	0.097	0.296	1.000
	Tl_BB versus TuR_MB	0.013	0.087	0.148	1.000

Note

Hazard ratios of infection by each fungus were estimated relative to the control within each species.

^*^ *p-value* < .05, ^**^ *p-value* < .01, ^***^ *p-value* < .001.

### 3.2 Intraspecific variation of spider mite susceptibility to infection by *Beauveria bassiana* and *Metarhizium brunneum*

**Figure 2:**
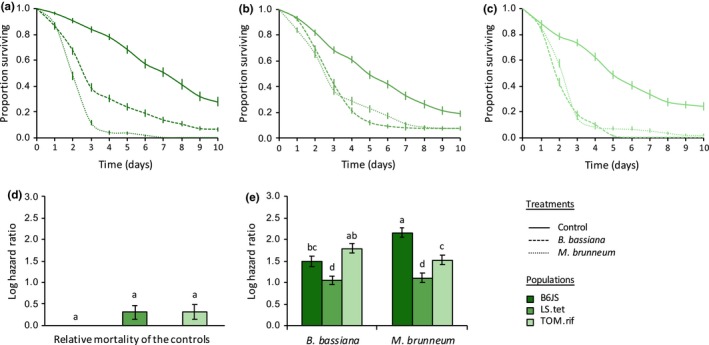
Survival curves of spider mite females (proportion surviving ± *SE*) from different populations of the green form of *Tetranychus urticae*: (a) B6JS, (b) LS.tet, and (c) TOM.rif, sprayed with *Beauveria bassiana* (dashed lines), *Metarhizium brunneum* (dotted lines), or with Tween‐20 only (control; solid lines). (d) Estimated mortality of the controls (sprayed with Tween‐20 only) of each spider mite population relative to the control of the population B6JS (log hazard ratio ± *SE*). (e) Estimated mortality of each spider mite population upon infection by each fungus relative to their respective control (log hazard ratio ± *SE*); identical letter superscripts indicate nonsignificant differences between treatments at the 5% level (multiple comparisons with Holm correction)

**Figure 3:**
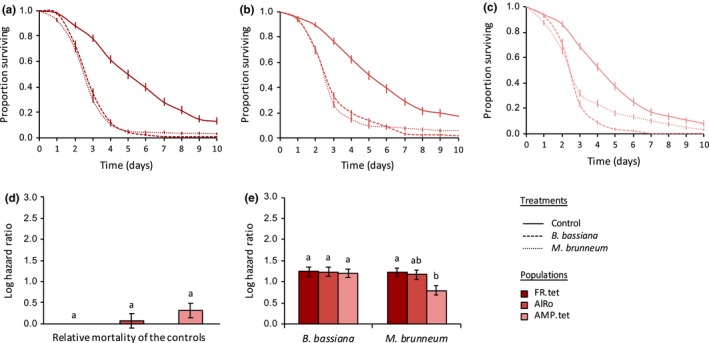
Survival curves of spider mite females (proportion surviving ± *SE*) from different populations of the red form of *Tetranychus urticae*: (a) FR.tet, (b) AlRo, and (c) AMP.tet, sprayed with *Beauveria bassiana* (dashed lines), *Metarhizium brunneum* (dotted lines), or with Tween‐20 only (control; solid lines). (d) Estimated mortality of the controls (sprayed with Tween‐20 only) of each spider mite population relative to the control of the population FR.tet (log hazard ratio ± *SE*). (e) Estimated mortality of each spider mite population upon infection by each fungus relative to their respective control (log hazard ratio ± *SE*); identical letter superscripts indicate nonsignificant differences between treatments at the 5% level (multiple comparisons with Holm correction)

**Figure 4:**
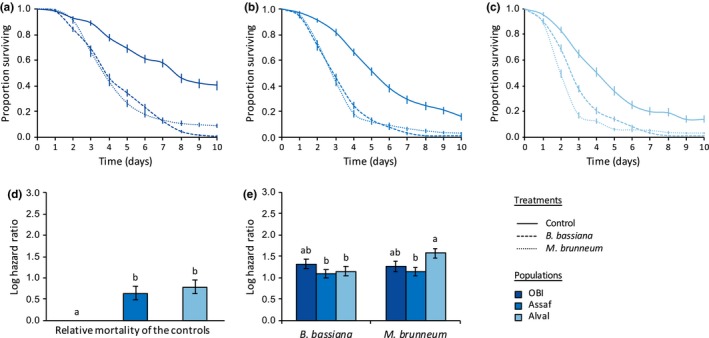
Survival curves of spider mite females (proportion surviving ± *SE*) from different *Tetranychus ludeni* populations: (a) OBI, (b) Assaf, and (c) Alval, sprayed with *Beauveria bassiana* (dashed lines), *Metarhizium brunneum* (dotted lines), or with Tween‐20 only (control; solid lines). (d) Estimated mortality of the controls (sprayed with Tween‐20 only) of each spider mite population relative to the control of the population OBI (log hazard ratio ± *SE*). (e) Estimated mortality of each spider mite population upon infection by each fungus relative to their respective control (log hazard ratio ± *SE*); identical letter superscripts indicate nonsignificant differences between treatments at the 5% level (multiple comparisons with Holm correction)

**Figure 5:**
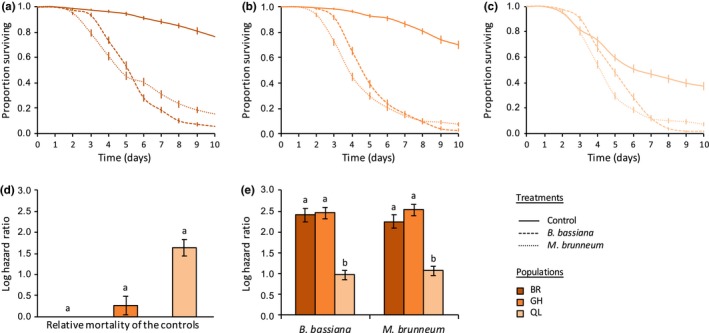
Survival curves of spider mite females (proportion surviving ± *SE*) from different *Tetranychus evansi* populations: (a) BR, (b) GH, and (c) QL, sprayed with *Beauveria bassiana* (dashed lines), *Metarhizium brunneum* (dotted lines), or with Tween‐20 only (control; solid lines). (d) Estimated mortality of the controls (sprayed with Tween‐20 only) of each spider mite population relative to the control of the population BR (log hazard ratio ± *SE*). (e) Estimated mortality of each spider mite population upon infection by each fungus relative to their respective control (log hazard ratio ± *SE*); identical letter superscripts indicate nonsignificant differences between treatments at the 5% level (multiple comparisons with Holm correction)

We also found a statistically significant interaction between infection treatment and population on spider mite survival within each of the species studied (in the green form of *T. urticae*: *X* ^2^_4_ = 79.60, *p* < .0001; Figure 2; in the red form *T. urticae*: *X* ^2^_4_ = 12.12, *p* < .02; Figure 3; in *T. ludeni*: *X* ^2^_4_ = 17.41, *p* < .002; Figure 4; in *T. evansi*: *X* ^2^_4_ = 106.72, *p* < .0001; Figure 5). Indeed, although the two fungi induced a similar mortality in most populations within each species (e.g., the populations LS.tet, FR.tet, AlRo, OBI, Assaf, and all populations of *T. evansi*; see Table 3 for the statistical results of all comparisons), *B. bassiana *induced a higher mortality than *M. brunneum* in the populations TOM.rif and AMP.tet (green and red form of *T. urticae*, respectively); and the reverse was found in the populations B6JS and Alval (green form of *T. urticae* and *T. ludeni*, respectively). Moreover, although the susceptibility to infection was relatively similar between populations of the red form of *T. urticae* (Figure 3e) and in *T. ludeni* (Figure 4e), we found important variation between populations of the green form of *T. urticae* (Figure 2e) and in *T. evansi* (Figure 5e). In the green form of *T. urticae*, *B. bassiana *induced a higher mortality in the populations TOM.rif and B6JS (HR = 6.01 and HR = 4.41, respectively) than in the population LS.tet (HR = 2.85; Figure 2e; Table 3a). Similarly, *M. brunneum* induced higher mortality in B6JS (HR = 8.67) than in TOM.rif (HR = 4.60), and the lowest mortality was found in LS.tet (HR = 3.04; Figure 2e; Table 3a). In *T. evansi*, both fungi species induced a higher mortality in the populations GH and BR than in the population QL (HR = 12.13, HR = 10.25, and HR = 2.77 in average, respectively; Figure 5e; Table 3d). Note, however, that QL control had a much lower survival than that of the two other populations (Figure 5d; Table 3d).

**Table 3:** Results of the multiple comparisons (with Holm correction) between hazard ratios obtained for different populations of (a) the green form of *Tetranychus urticae*, (b) the red form of *Tetranychus urticae*, (c) *Tetranychus ludeni*, and (d) *Tetranychus evansi* among the different treatments (BB: *Beauveria bassiana*; MB: *Metarhizium brunneum*; C: control)

Type of comparison	Populations_treatments compared	Estimate	*SE*	*z* value	*p*‐value
(a) Multiple comparisons between populations of the green form of *T. urticae*					
Between controls	TOM.rif_C versus LS.tet_C	−0.002	0.127	−0.018	1.000
	LS.tet_C versus B6JS_C	0.307	0.165	1.863	.312
	B6JS_C versus TOM.rif_C	−0.305	0.169	−1.809	.312
Between populations sprayed with *B. bassiana *	B6JS_BB versus TOM.rif_BB	−0.302	0.156	−1.981	.285
	B6JS_BB versus LS.tet_BB	0.436	0.155	2.813	0.044*
Between populations sprayed with *M. brunneum*	B6JS_MB versus TOM.rif_MB	0.634	0.154	4.118	<.0005***
	TOM.rif_MB versus LS.tet_MB	0.414	0.147	2.816	.044*
Between fungi within or among populations	TOM.rif_BB versus TOM.rif_MB	0.268	0.093	2.868	.041*
	LS.tet_BB versus LS.tet_MB	−0.065	0.096	−0.671	1.000
	B6JS_BB versus B6JS_MB	−0.676	0.099	−6.857	8.42e−11***
	B6JS_BB versus TOM.rif_MB	−0.042	0.156	−0.271	1.000
	TOM.rif_BB versus B6JS_MB	−0.366	0.153	−3.387	.119
(b) Multiple comparisons between populations of the red form of *T. urticae*					
Between controls	AlRo_C versus AMP.tet_C	−0.244	0.151	−1.621	.841
	AMP.tet_C versus FR.tet_C	0.309	0.162	1.903	.513
	FR.tet_C versus AlRo_C	−0.065	0.161	−0.404	1.000
Between populations sprayed with *B. bassiana *	AlRo_BB versus FR.tet_BB	−0.011	0.149	−0.072	1.000
	AlRo_BB versus AMP.tet_BB	0.037	0.147	0.248	1.000
	FR.tet_BB versus AMP.tet_BB	0.047	0.146	0.324	1.000
Between populations sprayed with *M. brunneum*	AlRo_MB versus FR.tet_MB	−0.062	0.148	−0.416	1.000
	AlRo_MB versus AMP.tet_MB	0.375	0.154	2.431	.151
	FR.tet_MB versus AMP.tet_MB	0.437	0.154	2.843	.049*
Between fungi within populations	AlRo_BB versus AlRo_MB	0.068	0.098	0.690	1.000
	AMP.tet_BB versus AMP.tet_MB	0.407	0.102	3.979	.0008***
	FR.tet_BB versus FR.tet_MB	0.017	0.094	0.182	1.000
(c) Multiple comparisons between populations of *T. ludeni*					
Between controls	Assaf_C versus Assaf_C	−0.639	0.156	−4.088	.0005***
	Alval_C versus Alval_C	−0.141	0.144	−0.980	1.000
	OBI_C versus OBI_C	0.780	0.158	4.945	9.91e−06***
Between populations sprayed with *B. bassiana *	Assaf_BB versus Assaf_BB	0.237	0.150	1.581	.867
	Alval_BB versus Alval_BB	0.171	0.155	1.101	1.000
	OBI_BB versus OBI_BB	0.066	0.147	0.447	1.000
Between populations sprayed with *M. brunneum*	Assaf_MB versus Assaf_MB	0.113	0.153	0.738	1.000
	Alval_MB versus Alval_MB	−0.315	0.159	−1.983	.426
	OBI_MB versus OBI_MB	0.428	0.150	2.858	.043*
Between fungi within or among populations	OBI_MB versus OBI_MB	0.062	0.096	0.645	1.000
	Assaf_MB versus Assaf_MB	−0.062	0.094	−0.654	1.000
	Alval_MB versus Alval_MB	−0.424	0.095	−4.469	9.42e−05***
	Alval_MB versus Alval_MB	−0.253	0.157	−1.606	.867
(d) Multiple comparisons between populations of *T. evansi*					
Between controls	BR_C versus GH_C	−0.263	0.218	−1.205	.914
	GH_C versus QL_C	−1.370	0.189	−7.236	3.70e−12***
	QL_C versus BR_C	1.633	0.201	8.145	4.44e−15***
Between populations sprayed with *B. bassiana *	BR_BB versus GH_BB	0.207	0.207	−0.254	.914
	BR_BB versus QL_BB	1.435	0.188	7.631	2.10e−13***
Between populations sprayed with *M. brunneum*	BR_MB versus GH_MB	−0.289	0.210	−1.375	.846
	BR_MB versus QL_MB	1.176	0.192	6.117	6.68e−9***
Between fungi within or among populations	BR_BB versus BR_MB	0.161	0.104	1.556	.718
	GH_BB versus GH_MB	−0.075	0.097	−0.775	.914
	QL_BB versus QL_MB	−0.098	0.094	−1.039	.914

Note

Hazard ratios of infection by each fungus were estimated relative to the control within each population.

^*^ *p-value* < .05, ^**^ *p-value* < .01, ^***^ *p-value* < .001.

## 4 DISCUSSION

In this study, we found both intra‐ and interspecific variability in the susceptibility of *Tetranychus* spider mite to infection by *B. bassiana *and *M. brunneum*. Overall, we observed a higher mortality upon infection in *T. evansi* and in the green form of *T. urticae*, than in *T. ludeni* and in the red form of *T. urticae*. These results, however, may not reflect accurately the virulence of both fungi in each of these spider mite species. Indeed, we further found important variation among populations within each species. Most variation was found among populations of *T. evansi* and of the green form of *T. urticae*, with, for instance, the mortality upon infection of two populations of *T. evansi* (BR and GH) being 5 times higher than that of another (QL). We also found variation among populations of *T. ludeni* and of the red form of *T. urticae*, although the amplitude of these effects was relatively smaller and depended on the fungal species.

Overall, our results suggest that spider mite susceptibility to infection is not a phylogenetically conserved trait, and further corroborate the generalist status of both fungal species (Meyling & Eilenberg, 2007; Rehner, 2005; Roberts & Leger, 2004). For instance, *B. bassiana *occurs naturally in more than 700 host species (Inglis, Goettel, Butt, & Strasser, 2001), and this range is likely underestimated as prevalence estimates are usually done in arthropod species that are crop pests or predators and parasitoids used as biocontrol agents (Meyling & Eilenberg, 2007). Moreover, differences in virulence between the two fungi shown here suggest population‐specific responses to each fungus, instead of a more general response against infection. For instance, *M. brunneum* is more virulent than *B. bassiana *in the population B6JS of green *T. urticae* and in the population Alval of *T. ludeni*, while the reverse occurred in the population AMP.tet of the red form of *T. urticae*. Such differences in susceptibility to infection between populations independently of their phylogenetic relationship may thus reflect differences in exposure by each fungus species (i.e., different selection pressure for resistance mechanisms to evolve) throughout their evolutionary history.

Variations in the prevalence of each fungus leading to different exposure may, for instance, occur between different geographical areas due to several environmental factors, such as temperature, humidity, and solar (UV) irradiation (Meyling & Eilenberg, 2007). However, these fungi are known to have a cosmopolitan distribution, and our results show no clear association between the susceptibility of a particular spider mite population and its country of origin. For instance, the *T. evansi* populations BR and GH come from Brazil and Portugal, respectively, but do not differ in susceptibility to infection by both fungi; similarly, the effect of *B. bassiana *does not differ between populations of red *T. urticae* collected in France (FR.tet), Spain (AlRo), and Portugal (AMP.tet). Instead, we found different susceptibility to infection between populations at small geographical scales, such as in the *T. evansi* populations GH and QL and in the green *T. urticae* populations B6JS and TOM.rif upon infection by both fungi or in the *T. ludeni* populations Assaf and Alval upon infection by *M. brunneum*, while all of these populations were collected in the same region in Portugal. These results might thus be explained by microhabitats‐specific distribution of the fungi, as previously found for different isolates of *B. bassiana *(e.g., Ormond, Thomas, Pugh, Pell, & Roy, 2010; Wang, Shah, Patel, Li, & Butt, 2003). Moreover, several studies suggest that both *B. bassiana *and *Metarhizium* spp. have the potential to interact directly with the host plants of arthropods (reviewed in Meyling & Eilenberg, 2007), which may potentially lead to plant‐specific distribution of the fungi. Indeed, *Metarhizium* spp. occur in the rhizosphere, which possibly provides a “refuge” where the fungus can survive outside insect hosts, and the presence of *B. bassiana *in internal plant tissue has been discussed as an adaptive protection against herbivorous insects (reviewed in Meyling & Eilenberg, 2007). However, the host plant range of these fungi is, to our knowledge, as yet unknown. Moreover, no field survey of these fungi has been conducted to date in *Tetranychus* spp. (but see, for instance, Dick & Buschman, 1995, Van Der Geest, Moraes, Navia, & Tanzini, 2002, for other fungi and/or spider mite species, Debnath & Sreerama Kumar, 2017). Future evaluation of the prevalence of infection by *M. brunneum* and *B. bassiana *in natural populations of spider mites collected on different host plants would thus be necessary to further understand possible factors that could explain the patterns observed in our experiment (Boots, Best, Miller, & White, 2009).

Decreased host susceptibility to infection may be the result of two different (albeit nonexclusive) mechanisms (Boots et al., 2009; Read, Graham, & Raberg, 2008): resistance (i.e., reduction in parasite load) and/or tolerance (i.e., reduction of the damage incurred by a parasite). Differential host resistance to fungal infection might be due, for instance, to variability in different cuticular barriers. Such barriers include the absence of factors necessary for parasite recognition, or the presence of inhibitory compounds (phenols, quinones, and lipids) on the cuticle surface, but also the cuticle thickness, its degree of hardening by sclerotization, its resistance to enzymatic degradation, and its permeability (reviewed in Hajek & St. Leger, 1994). Subsequently, when a fungus bypass cuticular barriers, variability in systemic immunity may also lead to differential host resistance responses. This may include differential activation of the Toll and JAK/STAT pathways, which converge into the transcriptional activation of genes involved in phagocytosis, encapsulation, and humoral responses (e.g., Dong, Morton, Ramirez, Souza‐Neto, & Dimopoulos, 2012). Interestingly, several, but not all, important genes described in these pathways in *Drosophila melanosgaster* were absent in the genome of the green form of *T. urticae* (Grbic et al., 2011), and spider mites have high mortality upon bacterial infection (Santos‐Matos et al., 2017). Whether the presence of such immune genes varies between or within spider mite species and whether their expression depends on different fungal species have not been explored to date. In particular, the absence of many important immune genes in *T. urticae* suggests that tolerance mechanisms (e.g., via a decrease of the immune response to avoid autophagy) rather than resistance have been favored throughout their evolutionary history. However, such hypothesis remains to be tested and further studies are necessary to better understand the mechanisms of spider mite resistance and tolerance against fungal infection.

Independently of the underlying mechanisms at play, whether spider mite populations differ in resistance or tolerance to fungal infection may have different epidemiological and evolutionary consequences, and, hence, different implications for the long‐term success of spider mite control. On the one hand, resistance to infection might be rapidly selected following application of fungi to crops and subsequently invade spider mite populations, thereby decreasing fungi prevalence and hampering the success of such control strategy. On the other hand, host tolerance should have neutral or even positive effect on parasite prevalence (Boots et al., 2009; Miller, White, & Boots, 2006; Read et al., 2008), but as, by definition, tolerance minimizes the harm caused by pathogens, it may hamper the efficiency of fungi in controlling spider mites. Moreover, host resistance and tolerance may lead to different evolutionary outcomes for parasite virulence (Boots et al., 2009). Indeed, whereas host resistance is predicted to select for increased parasite virulence (e.g., Gandon & Michalakis, 2000), host tolerance does not reduce parasite fitness and, therefore, will not lead to antagonistic counter‐adaptation by pathogens (Raberg, Sim, & Read, 2007; Rausher, 2001). Still, depending on the nature of the tolerance mechanism, it may lead to the evolution of more virulent and transmissible parasites (Miller et al., 2006), with potentially serious implications for nontolerant populations (Boots et al., 2009), including nontarget species such as crop auxiliaries or spider mite predators. Finally, although increased mortality due to infection should lead to a reduction in oviposition duration, spider mites may evolve the ability to compensate infection‐driven fitness costs by changing the timing of their reproductive efforts (i.e., "fecundity compensation"; Parker, Barribeau, Laughton, Roode, & Gerardo, 2011; Vezilier, Nicot, Gandon, & Rivero, 2015), thereby limiting the efficiency of fungi applications for population control. Hence, assessing which of these evolutionary outcomes is more likely is timely. In particular, it is likely that the level of intraspecific variation in susceptibility to infection found in our study is recapitulated within populations and is, at least partly, genetically determined. If this is the case, then this trait may evolve at a rapid pace.

In conclusion, our results show both intra‐ and interspecific variability in spider mite susceptibility to fungi‐induced mortality using two generalist fungi, *B. bassiana *and *M. brunneum*. To our knowledge, this is the first study investigating the effect of entomopathogenic fungi on the survival of multiple spider mite populations belonging to different species within a single full factorial experiment. In line with laboratory virulence tests that are not necessarily well correlated with field effectiveness (Roberts & Leger, 2004), our results highlight the importance of studying several host populations/genomes when assessing the efficiency of a given biocontrol agent. These results also draw caution on the development of single strains as biocontrol agents, as hosts resistance to infection may evolve at a rapid pace.

## CONFLICT OF INTEREST

None declared.

## AUTHOR CONTRIBUTIONS

FZ and SM conceived and designed the experiment. IS was involved in the maintenance of spider mite populations and plants. MA acquired the data. FZ performed the statistical analysis. FZ and SM wrote the manuscript, with input from all authors. IC and SM funded the study. All authors have read and approved the final version of the manuscript.

## Data Availability

Full dataset has been deposited in the Dryad Data Repository (https://doi.org/10.5061/dryad.gmsbcc2j4).
